# Identification of promising alfalfa varieties in conditions of the southern forest-steppe zone (Republic of Bashkortostan): a study of economic and biological characteristics

**DOI:** 10.1186/s40529-022-00361-w

**Published:** 2022-10-22

**Authors:** Igor Kuznetsov, Ilgiz Asylbaev, Alexey Dmitriev, Asiya Nizaeva, Anvar Shakirzyanov

**Affiliations:** 1 Department of Plant Growing, Plant Breeding and Biotechnology, Federal State Budgetary Educational Establishment of Higher Education “Bashkir State Agrarian University”, Ufa, Russian Federation; 2 Department of Soil Science, Agrochemistry and Precision Farming, Federal State Budgetary Educational Establishment of Higher Education “Bashkir State Agrarian University”, Ufa, Russian Federation; 3 Bashkir Research Institute of Agriculture-A Separate Structural Division of the Federal State Budgetary Scientific Institution of the Ufa Federal Research Center of the Russian Academy of Sciences, Ufa, Russian Federation

**Keywords:** Legumes, Alfalfa, Variety testing, Seed productivity, Breeding, Forest-steppe

## Abstract

**Background:**

Alfalfa (*Medicago sativa* L.) is one of the most important forage crops, but its seed productivity varies from year to year due to weather conditions. Alfalfa is a forage crop rich in nutrients which makes it valuable. The present 5-year study aimed to identify the promising alfalfa varieties based on their economic and biological characteristics, such as high seed productivity, a high yield of forage mass, good quality, and stress resistance.

**Results:**

The study contributes information about the promising varieties of alfalfa characterized by high biomass and seed productivity. Varieties such as P-88044, U-73+149 and S-302 had stable seed/green mass yields regardless of climatic conditions; hence, they may be used for alfalfa selection in the southern forest-steppe regions of Bashkortostan.

**Conclusions:**

The present findings can be used for alfalfa selection in the Republic of Bashkortostan (Russia). In addition, the results will be of interest to crop breeders across the globe.

## Introduction

Alfalfa (*Medicago sativa* L.) or lucerne is one of the oldest and most widespread forage crops. It became widely known and favoured for its properties and economic value. Alfalfa is a highly nutritious and thus practical legume. Its forage mass during the blooming period equals 21–23 feed units (per 100 kg). The amount of digestible protein ranges from 4.05 to 4.12 kg (per 100 kg), 6.1–6.3 times more than in soybean (Kosolapov 2014). The leaves of this plant deserve special attention, for they make up 41 to 59% of its total weight. The percentage of crude protein in alfalfa leaves can reach 30%. Alfalfa hay and green mass are rich in amino acids, minerals and vitamins (Zhuchenko 2000; Safonov 2022). This perennial legume has great biological potential, sparking interest among breeders (Ignatiev and Regidin 2019; Ventsova and Safonov 2021).

## Literature review

According to research conducted in France, damaged seed lots require moderate temperatures to germinate (Ghaleb et al. 2021; Safonov et al. 2021). Climate variability, however, can impose high risks on lucerne production (Humphries et al. 2021). To solve this problem, some scientists created new hybrids between M. Sativa, M. Arborea L. (woody shrub), and M. Truncatula Gaertn. (an annual species from the Mediterranean region), but it also expanded genetic diversity (Humphries et al. 2021).

According to Kuznetsov et al. (2020), meteorological factors can also affect the growing season duration and grain yield capacity of different crop varieties.

Due to the lack of a reference genome and an effective genome editing protocol, it is hard to improve lucerne artificially. The main issue here is that alfalfa is a tetraploid and self-incompatible plant; in other words, it is allogamous. Mutated alleles and phenotypes of null mutants can be stably inherited by cross-pollination without using a transgene, which helps avoid disputes over transgenic plants (Chen et al. 2020).

Tlahig et al. (2021) introduced alfalfa genotypes, which can adapt well to arid environments outside the oasis. Samples of alfalfa from Iran proved to be resistant to drought and rainfall; these traits can help breed new enhanced varieties (Riasat et al. 2020). Some authors found a near-complete and accurate diploid alfalfa reference genome, which provides valuable genomic resources for improving breeding strategies in alfalfa (Li et al. 2020). Another challenge in creating synthetic varieties is the breeding of clear alfalfa lines, but the generation of phenotype-aligned micro populations could be a solution (Zhuchenko 2012).

Farmers who seek to enhance seed productivity in alfalfa may want to focus on the quantitative and species composition of insect pollinators (Dyukova et al. 2013). The Siberian Research Institute of Plant Selection and Breeding has experimentally created some highly productive varieties, but the sourcing of material was limited to Siberia (Goncharov et al. 2011). Other researchers isolated male-sterile plants. For this, a mitochondrial DNA marker was mapped and correlated with phenotypic traits of the plants (Ahmad et al. 2020).

One of the most acute problems in alfalfa-growing is the generation of stable (less altered) seed yields (Khasanov et al. 2019, 2020). It becomes most apparent in regions where climatic conditions change dramatically, inhibiting high alfalfa yields. These are continental climate regions. Examples include the interior regions of the United States, Canada, Russia, Central Asia and Mongolia. They make up a significant part of the land, up to 30% of the total area. Therefore, it seems relevant to seek varieties of alfalfa that produce seeds resistant to external influences and high biomass. Another region with a dominating continental climate is the Republic of Bashkortostan. Hence, it may be of interest as a model. The results obtained here can potentially apply to other continental climate regions. The study sample comprises 10 varieties of alfalfa, commonly grown in continental climate regions, which are resistant to drought and frost: Bibinur, U-73+149, U-964, S-302, C-344, P-85044, Camellia, Selena, Lugovaya 67, and Vega 87. The biometric indicators explored in this study include the green mass yield, seed productivity, plant height, leafiness, and flowering time.

Alfalfa has a high potential for seed productivity. It is stress-resistant and has a high yield of good-quality forage mass. Therefore, it is reasonable to cultivate alfalfa varieties and populations in the Republic of Bashkortostan (i.e., a model continental climate region).

The present study aims to identify the promising varieties of alfalfa based on some economic and biological characteristics (i.e., high seed productivity, a high yield of forage mass, good quality, and stress resistance). The objectives of the study are (1) to examine the biometric properties of alfalfa samples; and (2) to measure the green mass yield and seed productivity of various alfalfa varieties. The observation period was 5 years (2016–2020).

## Methods

### Soil description

The cultivation was performed on leached chernozem sites, which belong to the Bashkir Research Institute of Agriculture of the Russian Academy of Sciences (the southern forest-steppe region of Bashkortostan; single-species crop production). The soils here are heavy loamy soils (humus, 8.3–8.5%; mobile potassium, 125–128 mg/kg; phosphorus, 103–109 mg/kg) with a medium thickness. The phosphorus and potassium concentrations were analyzed using the Kirsanov method. The soil pH ranges from 6.1 to 6.3, indicating a neutral reaction of the soil environment.

### Climate description

According to the weather station Ufa-Dema, the average annual precipitation during the growing season (May + 1st decade of September) is 278 mm. The average daily air temperature is + 15.2 °C. The hydrothermal coefficient (GTC) is 1.22.

### Agricultural operations

The cultivation process of alfalfa relied on conventional techniques. Basic cultivation was carried out at a depth of 25 cm and combined with the application of phosphorus–potassium fertilizers R_90_K_60._ The early spring harrowing and pre-sowing cultivation (with harrowing) processes took place in spring. Seed rolling was carried out either before or after sowing. The sowing was done at the seed rates of 12 kg/ha (for green mass) and 3.5 kg/ha (for seeds) using an SN-16 seeder. The row-width spacing was 60 cm. The registration plot area was 50 m^2^ and had a fourfold design. The green mass was cut between the budding and early flowering stages. The seeds were harvested when 74 to 81% of the beans got brown. The cultivation process involved five complex hybrid populations obtained from open pollination of the best locally-adapted hybrids made by the Research Institute of Feed, the All-Russian Institute of Genetic Resources and other scientific institutions. The Bibinur variety of locally-adapted alfalfa was used as a reference (rf).

## Results

### Green mass yield, seed productivity, plant height, leafiness, and flowering time of alfalfa

The first varieties to enter the early flowering stage (accompanied by mowing ripeness) were U-964, C-302, C-344, P-85044, and Camellia. At the onset of the flowering stage, the plant height ranged from 86.8 to 88.7 cm. All samples were shorter than the reference one. Four varieties had a high ratio of leaf area (Table 1), namely P-85044 (56.3%), C-302 (56%), Camellia (55.8%) and C-344 (56.0%).

**Table 1:** Biometric indicators of alfalfa varieties

Subitem no.	Varieties	Onset date of flowering	Plant height at the onset of flowering, cm	Leaf area ratio, %
1	Bibinur (rf)	16.06	88.7	53.1
2	U-73+149	15.06	87.9	55.0
3	U-964	14.06	88.6	54.7
4	C-302	14.06	87.4	56.0
5	C-344	14.06	86.9	55.6
6	P-85044	14.06	88.6	56.3
7	Camellia	14.06	86.8	55.8
8	Selena	14.06	87.5	54.9
9	Lugovaya 67	14.06	87.9	55.1
10	Vega 87	14.06	88.1	55.3

Green mass and dry matter productivity are characteristics essential for creating a new breeding alfalfa material with an increased yield of forage mass. The varieties under study had varying green mass yields throughout the observation period. Due to drought in 2016, the 1-year-old alfalfa plantation failed to unlock its full potential for green mass and seed yield. Hence, only one cutting was done (Hydrothermal index, 0.78).

### Alfalfa yield and weather relationships

Weather conditions in 2017 positively affected the forage mass formation (Hydrothermal index, 1.5). The average daily air temperature in 2018 was 1.8 °C lower than the climatic norm (Hydrothermal index, 1.0). The precipitation level in the second half of May was 23 mm higher than the average annual precipitation, facilitating a high forage mass yield. The year 2019 saw a sufficient precipitation level and a relatively low air temperature (Hydrothermal index, 1.3). 2020 witnessed an uneven distribution of heat and moisture (Hydrothermal index, 1.19). In May, there was a shortage of precipitation—11 mm, with a norm of 47 mm; the temperature was 15.4 °C. The soil moisture deficit was present until the end of August and led to a slight decrease in the green mass during the second cutting. Over the 5-year observation period, the maximum green mass yields were recorded with varieties P-85044, C-302, C-344, U-73+149, Lugovaya 67, and Vega 87. These yields were 13.7, 11.3, 10.0, 9.8, 10.2, and 10.8% higher than the reference yield (Table 2).

**Table 2:** The green mass yields of alfalfa varieties, t/ha

No. item number	Varieties	Yield (green mass)					Total mean, t/ha	Yield (gain)	
		Years of research						t/ha	%
		2016	2017	2018	2019	2020			
1	Bibinur (rf)	12.3	43.9	32.6	38.8	30.2	31.56		
2	U-73+149	14.1	44.5	39.6	43.9	31.2	34.66	3.1	9.8
3	U-964	13.2	45.2	38.4	44.2	30.8	34.36	2.8	8.9
4	C-302	13.8	45.8	39.4	44.3	32.3	35.12	3.6	11.3
5	C-344	13.4	45.6	39.2	44.5	30.9	34.72	3.2	10.0
6	P-85044	14.3	46.2	39.2	44.7	35.0	35.88	4.3	13.7
7	Camellia	14.1	45.2	39.1	43.2	31.8	34.68	3.1	9.0
8	Selena	13.6	44.1	39.1	43.1	30.8	34.83	3.3	9.5
9	Lugovaya 67	13.9	44.6	39.2	43.7	31.4	34.67	3.5	10.2
10	Vega 87	13.8	45.2	39.3	43.6	33.1	34.91	3.9	10.8
Least significant difference_05_		0.06	0.08	0.05	0.09	0.08			

The weather appeared to have a significant impact on seed productivity in alfalfa. All varieties had the highest yield in 2019. At the same time, the summer drought in 2016 and 2018 resulted in reduced seed productivity. Excessive soil moisture during the growing season in 2017 (above 75% of water-holding capacity) and a small sum of effective temperatures delayed the flowering phase and boosted sprout formation and tillering. The consequences for alfalfa seed formation were negative—the varieties yielded no seeds. In 2020, the average daily temperatures did not reach the required minimum (20 °C) during the flowering phase. Conditions were only favourable in the second half of the first decade and in the third decade of July. The second decade of July saw a daytime temperature above 32 °C. Note that high temperatures affect the wild bees. With a temperature higher than 29 °C, the wild bees become less active; they stop flying completely when the temperature rises to 32 °C and above. During the observation period, P-85044 (− 0.245 t/h) and Vega 87 exhibited the highest seed productivity, which exceeded the reference value by 17.4 and 16.2%, respectively (Table 3).

**Table 3:** The seed yields of alfalfa varieties, t/ha

No. item number	Varieties	Yield (seeds)					Total mean, t/ha	Yield (gain)	
		Years of research						t/ha	%
		2016	2017	2018	2019	2020			
1	Bibinur (rf)	0.114	–	0.100	0.527	0.301	0.208		
2	U-73+149	0.127	–	0.125	0.620	0.318	0.238	0.030	14.2
3	U-964	0.125	–	0.112	0.614	0.316	0.233	0.025	12.0
4	C-302	0.126	–	0.130	0.623	0.315	0.239	0.030	14.6
5	C-344	0.123	–	0.128	0.618	0.311	0.236	0.028	13.2
6	P-85044	0.123	–	0.132	0.635	0.333	0.245	0.036	17.4
7	Camellia	0.129		0.130	0.610	0.307	0.235	0.027	12.9
8	Selena	0.126	–	0.116	0.615	0.317	0.232	0.030	14.7
9	Lugovaya 67	0.127	–	0.121	0.632	0.322	0.238	0.032	15.7
10	Vega 87	0.125	–	0.125	0.621	0.330	0.241	0.033	16.2

## Discussion

The results of the present study are consistent with previous research conducted in the Yakutsk suburbs. Atlasova (2009) examined 50 alfalfa samples, and some of them had a high seed yielding capacity exceeding the reference value (Yakut yellow variety) by 24.5 to 36.1%. Among them are samples 3 (0.82 c/ha), 16 (0.81 c/ha) and 47 (0.82 c/ha) as well as variety Syulinskaya (0.9 c/ha).

At the same time, Luz et al. (2020) point out that the production of hybrid seeds is now highly inefficient. The reason is the use of lines obtained by successive self-fertilization. Therefore, it is necessary to look for alternatives to reduce the effect of line inbreeding depression. The authors performed amplifications using eight microsatellite primers with high polymorphism. Based on the results, they proposed to obtain hybrids between different combinations (Luz et al. 2020).

Tormozin and Zyryantseva (2019) from the Ural Research Institute of Agriculture found the varieties Victoria, SGP-1, SGP-2, and 193-95d to be rather promising. These varieties exhibited good results and significantly exceeded the seed yield of the Sarga variety (reference) by 25, 39, 93, and 61%, respectively. This finding suggests that these varieties have a high adaptive potential.

Mamalyga et al. (2020) investigated the parent material resistant to high soil acidity and used it to synthesize new-generation alfalfa varieties. The researchers examined 30 varieties harvested in various geographical zones of Russia. The research showed that the Ferax 58 variety had the highest adaptive potential due to its genetic characteristics. Radoslava, Olga, and Vavilovka varieties have also given a good account of themselves. Farmers can utilize those as donors for creating highly productive varieties or hybrid populations resistant to elevated soil acidity (Mamalyga et al. 2020).

Some studies related to alfalfa breeding were conducted at the Yershov experimental station. This research has been at the forefront at the station since 1976. According to Popova (2020), more than 1.500 samples have been sown throughout this research period, including 1.430 samples obtained at N.I. Vavilov Research Institute of Plant Industry. An artificial crossing of 790 hybrid combinations was carried out. Each year, researchers at the station sow 10 to 15 population samples for competitive testing. According to the experiments (1917–1919), populations No. 5/12, 5/10, 5/08, and 6/14 had high seed and green mass yields. Some researchers examined the collection of agricultural plants from the All-Russia Institute of Plant Genetic Resources. In terms of ripeness, the varieties from France and Hungary outperformed other specimens in that collection. Meantime, those from Sweden and the USA had better seed parameters (Popova 2020).

According to Xu et al. (2021), the production of haploid and double haploid plants is among China’s most famous breeding directions. The said researchers exposed haploid plants to different colchicine concentrations. The result was that three explants showed a doubling effect (Xu et al. 2021).

Farmers in a continental climate region with cold and dry winters previously reported achieving good alfalfa yields with phosphorus fertilizers; in these cases, alfalfa was planted in late summer (Wang et al. 2022). In Kazakhstan, high alfalfa and seed yields were achieved through the combined use of insecticides and fertilizers; grey soils under which they were cultivated underwent conventional treatment (Absatova et al. 2022). In Turkey, farmers achieved high hay and seed yields even later, in early October (Kir 2022). The Swedish climate allows farmers to obtain high alfalfa yields through intercropping legumes and grasses (Mårtensson et al. 2022).

When cultivated in Bashkorkostan, alfalfa can be highly productive. The natural and climatic conditions of the country are conducive to better alfalfa production, making it possible to unlock the genetic potential of locally-adapted domestic and new varieties for high productivity. The same effect can be achieved in other continental climate regions. Alfalfa varieties such as P-85044 and Vega 87 had high seed yields; therefore, one can recommend these varieties for cultivation in continental climate regions. Alfalfa varieties with the fastest growing leaves were P-85044, C-302, Camellia and C-344. The green mass yield was found to be the highest in P-85044, C-302, C-344, U-73+149, Lugovaya 67, and Vega 87. These findings suggest that in Bashkorkostan, farmers can grow most of the studied varieties and expect good yields of green mass. In this case, 50% of those varieties will have good leaf growth, but only 20% will offer a high seed yield. Consequently, seed productivity should be considered a priority indicator when selecting alfalfa varieties for cultivation in continental climate regions; otherwise, a high seed yield will not be achieved. Leaf growth holds less significance as a selection criterion. In large-scale cultivation conditions, it is best to use varieties of alfalfa bred and propagated in similar climatic conditions. In the present case, such varieties include those from the Ural and Siberian selection.

Overall, agricultural plant breeders in Russia now face an acute dilemma. It is either free re-pollination of the best locally-adapted hybrids or gene modification. This problem is also relevant for scientists in other countries.

## Conclusions

Studies conducted in the 2016–2020 period suggest which alfalfa varieties are the most promising ones from the cultivation perspective. Criteria for selecting the best option were a high yield of forage mass and good seed productivity. For the southern forest-steppe region in Bashkortostan, the best choices would be varieties P-88044, U-73+149 and C-302, which exhibited climate-change resistance and a high seed/green mass yielding capacity. The present findings will be helpful to researchers in the Republic of Belarus, Russia, and other continental climate regions.

## Data Availability

The datasets generated during and/or analyzed during the current study are available from the corresponding author upon reasonable request.
